# Systemic and Oral Factors Relating to Stress in Later Life: A Study Using the Japanese National Statistics Database

**DOI:** 10.3390/clinpract15120226

**Published:** 2025-12-01

**Authors:** Kanako Seino, Akira Komatsuzaki, Kanako Mitomi, Mio Susuga, Sachie Ono, Yukika Enoki, Asami Iguchi, Hiromi Fujita, Naru Komatsuzaki

**Affiliations:** 1 Department of Dental Hygiene, The Nippon Dental University College at Niigata, 1-8 Hamaura cho, Chuo-ku, Niigata 951-8580, Japan; springsea@ngt.ndu.ac.jp (K.S.); hsjc@ngt.ndu.ac.jp (M.S.); enoki11@ngt.ndu.ac.jp (Y.E.); fussa@ngt.ndu.ac.jp (H.F.); 2 Department of Preventive and Community and Dentistry, School of Life Dentistry at Niigata, The Nippon Dental University, 1–8 Hamaura-cho, Chuo-ku, Niigata 951-8580, Japan; sachie@ngt.ndu.ac.jp; 3 Department of Dental Technology, The Nippon Dental University College at Niigata, 1-8 Hamaura cho, Chuo-ku, Niigata 951-8580, Japan; kmitomi@ngt.ndu.ac.jp (K.M.); tandai101@ngt.ndu.ac.jp (N.K.); 4 Department of Dental Anesthesiology, School of Life Dentistry at Niigata, The Nippon Dental University, Chuo-ku, Niigata 951-8580, Japan; asami@ngt.ndu.ac.jp

**Keywords:** stress, national statistics, subjective symptoms, diseases requiring hospital visits, older adults

## Abstract

**Background**: The psychosomatic effects of stress are risk factors for a range of dental and systemic diseases. This study used the massive Japanese national statistics database to clarify the association of psychological stress with subjective symptoms and conditions requiring hospital visits. **Methods**: Anonymized data from 93,690 respondents of the 2019 Japanese survey were provided for this study. From these data, those of 29,777 respondents aged 40–89 years were classified into stress groups based on their responses to the Kessler Psychological Distress Scale (K6). The response rates for symptoms and diseases were compared and analyzed using contingency tables and binomial logistic regression. **Results**: The items with the largest odds ratios in the univariate analysis were depression/other mental disease (7.60), followed by irritability (6.86) and health perception QOL (6.31). Among those with subjective symptoms, the proportion in the high-stress group was higher (15.1%), with a univariate odds ratio of 3.17. The results of the binomial logistic regression analysis, with subjective QOL as the dependent variable, were as follows: The explanatory variables with the highest adjusted odds ratios were stress group classification (3.14), followed by feeling physically tired (2.44) and sleep satisfaction (2.22). The stress group was associated with subjective symptoms, such as irritability and depression/other mental diseases, as well as with social factors, such as household structure and work hours. These results suggest the existence of specific symptoms, diseases, and environmental factors associated with high stress. **Conclusions**: The results suggest that stress may have a substantial impact on quality of life in later life. Therefore, healthcare measures for older adults should focus on the symptoms and diseases that tend to be associated with stress to mitigate their effects.

## 1. Introduction

According to the World Mental Health Survey published by the World Health Organization (WHO) in 2016, there is a high global prevalence of mental disease, and in some countries, such as the United States, it is in excess of 20% [1]. In Japan, there are many affected individuals as well. The number of patients with mental disease was approximately 3 million in 2005, which rapidly grew to 6.14 million by 2020 [2]. The number of people experiencing stress has also increased, which is a major risk factor for physical and mental disease [3]. In particular, suicide among older adults has become a major societal problem in Japan [4]. At the international level, it has been shown that countries with high numbers of suicides among older adults tend to rank high in global suicide rates; thus, countermeasures are needed to address this issue [5].

Moreover, stress is not only a risk factor for mental diseases such as depression [6] but also a range of physical health problems and systemic diseases [7], as well as oral diseases, such as periodontal disease [8]. In addition to physical effects, stress exerts a large toll on society. The gross domestic product (GDP) losses due to stress have been estimated at approximately 1% of GDP [9]. Mental health is a fundamental requirement for ensuring quality of life (QOL), as shown by its inclusion in the WHO’s definition of health [10]. Additionally, the relationship between the entire body and oral function is attracting attention. Researchers are analyzing the relationship between stress and periodontal disease and temporomandibular joint disease [11].

Mental health is affected by many factors, which include an individual’s stress-coping behaviors, their physical condition, the adequacy of their rest, and social factors [12]. Hence, while infectious diseases, for example, can be addressed using straightforward measures that target the cause, this approach is not readily applicable to mental health. The effects of stress on the mind and body have long been shown to be related to specific symptoms and conditions, as indicated by the use in Japan of the term “psychosomatic disease” [13,14]. Basic research has shown that the burden of stress on the immune system makes people more susceptible to infectious diseases [15] and that certain personality traits predispose people to cardiovascular disease [16].

The recognition and evaluation of symptoms can greatly vary depending on the individual’s subjective perception; consequently, it is clear that some conditions are more susceptible to the effects of stress than others [17]. Prior research by the present authors showed that stress is related to musculoskeletal system symptoms, such as “feeling listless,” and to specific diseases that require hospital visits, such as high blood pressure [18].

Meanwhile, critical reports have also acknowledged that the effects of stress on the mind and body involve multiple factors, as well as individual differences, such as personality [19], environmental factors [20], and social factors, such as lack of social isolation [21]. Therefore, this study also adopted variables related to household structure and employment.

In this study, we used a large-scale database to investigate the relationship between stress and subjective symptoms and diseases requiring hospital visits to clarify the processes whereby stress impacts QOL during old age. This descriptive epidemiological study in older subjects, who are most at risk from the effects of the need for nursing care, aimed to clarify the related factors, such as subjective symptoms, influencing body frailty and QOL.

## 2. Subjects and Methods

### 2.1. Study Design and Subject Data

**Figure 1:**
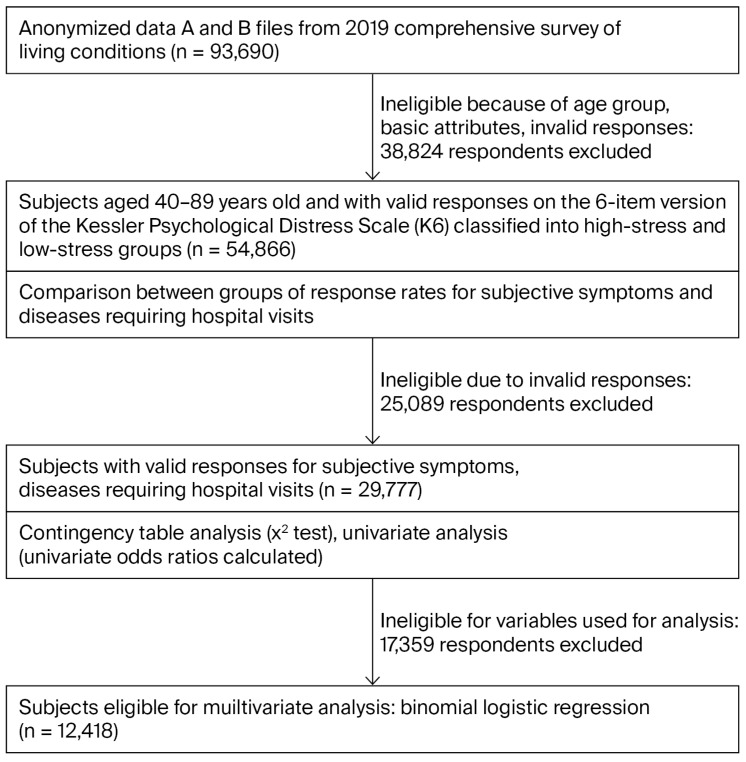
Outline of the data analysis.

This study utilized a cross-sectional design. The Ministry of Health, Labor, and Welfare (MHLW) of Japan has an established service for the provision of anonymous data from the national statistics for scientific research based on the Statistics Act, and it makes individual questionnaires that have been processed available to ensure they are anonymized. The authors obtained the anonymized data from the combined A and B files of the Comprehensive Survey of Living Conditions, corresponding to 93,690 people, with the MHLW’s permission, and selected subjects for analysis according to the stepwise process shown in Figure 1. Subjects were excluded from the data extraction process due to being outside the age range of this study or having missing data at each stage of the analysis.

Because this study focused on the old age group (≥65 years), the subjects for analysis were selected from those in the age range of 40–89 years (control group: 40–64 years). In Japan, people aged 40 and over are required to enroll in the long-term care insurance program. Many adult health checkups and activities aimed at preventing lifestyle-related diseases also target this age group. In this study, the elderly group (65 and over) was compared with a control group of people aged 40 to 64.

Using the results of the 6-item version of the Kessler Psychological Distress Scale (K6), respondents were classified based on the criteria of Komatsuzaki et al. [22] into a high-stress group (4864 individuals) and a low-stress group (50,002 individuals). These groups were used for the analyses detailed below (shown in Table 1).

### 2.2. Comparison Based on Stress Group of Responses Regarding Symptoms and Diseases, and Contingency Table Analysis (Univariate Analysis)

Differences in ranking between groups were compared using the Wilcoxon signed-rank test to compare the response rate rankings of symptoms and diseases by stress group. Mean values of reported symptoms were compared between the stress groups using Welch’s *t*-test. In addition, a contingency table was constructed for a univariate analysis of stress group and basic attributes, including gender, age, and work hours, as well as survey items such as subjective symptoms, diseases requiring hospital visits, and health awareness (a subjective sense of well-being, considered as QOL). Differences in response rates were compared using a X^2^ test, and the univariate odds ratios (ORs) were calculated.

### 2.3. Analysis of Effect on QOL Using Multivariate Analysis

Symptoms and diseases that were shown in the contingency table to be associated with the stress group were analyzed using binomial logistic analysis (the complete enumeration method). The category settings for each explanatory variable are shown in Tables 4–7. In Model 1 of the binomial logistic analysis, the stress group was set as the dependent variable, with gender and work time included as moderator variables, and the ORs were calculated. In Model 2, subjective sense of well-being (QOL) was set as the dependent variable, with stress group and the explanatory variables from Model 1 as explanatory variables.

### 2.4. Statistical Analysis

The dataset was extracted from all items. Categorical data were described in terms of counts and percentages. Quantitative data were categorized and used similarly for analysis using contingency tables. Statistically significant estimates allowed us to utilize the appropriate chi-squared (X^2^) tests to assess relationships and differences between groups.

Basic data aggregation was carried out using Excel 2019 (Microsoft Japan, Tokyo, Japan) and BellCurve for Excel-2019MSO statistical analysis software (BellCurve, Tokyo, Japan). The following methods were used to confirm statistical significance: the X^2^ test, the calculation of univariate odds ratios (ORs), the Wilcoxon signed-rank test, and binomial logistic regression analysis. The level of significance for all statistical tests was set at *p* < 0.05.

### 2.5. Ethical Considerations

This study was based on anonymized data files from national statistics. The authors received data files in tabular form that were provided by the MHLW following anonymization, and the data comprised only responses from respondents who consented to the anonymous database requirements. We did not perform additional processing, as data collection by the MHLW was anonymous and non-interventional. This study was conducted per the principles of the Declaration of Helsinki statement and Japanese Ethical Guidelines for Medical and Health Research Involving Human Subjects (MHLW, formulated on 23 March 2021) [23].

This study was approved by the Ethical Review Board of the Nippon Dental University College at Niigata (approval no.: NDUC-127). The Japanese large-scale database used for this study contains only anonymized data. The study protocol was pre-inspected and approved by the MHLW (Government Statistics 0805 No. 3), as stipulated by Article 36 of the Statistics Act.

## 3. Results

### 3.1. Responses to the Six-Item Version of the Kessler Psychological Distress Scale: K6 and Stress Group Classification

Responses to the K6 are shown in Table 1. There were 21,692 (39.5%) respondents with a K6-score of 0, accounting for approximately 40%, while 67.1% of the respondents scored ≤ 3. The high-stress group, which comprised subjects with a score of ≥10 (*n* = 4864), accounted for 8.9% of the total.

### 3.2. Comparison by Stress Group of Response Rate Ranking and Mean Number of Responses for Subjective Symptoms and Diseases Requiring Hospital Visits

Table 2 and Table 3 show the top 10 ranking responses for subjective symptoms and diseases requiring hospital visits. In both the high-stress and low-stress groups, the highest-ranked subjective symptoms were musculoskeletal symptoms, including lower back pain, stiff shoulders, and arm/leg joint pain, while the top-ranked symptoms in the high-stress group also included feeling listless, sleeplessness, and irritability. For diseases requiring hospital visits, depression/other mental disease (16.1%) was ranked third in the high-stress group; otherwise, almost the same diseases were included in the top-10 rankings for both the high- and low-stress groups.

Dental symptoms were present in the top 10 response rate rankings for both the high- and low-stress groups, ranking fourth in the low-stress group. Regarding the diseases requiring hospital visits, dental disease was ranked fifth in both the high- and low-stress groups.

The mean response rate rankings of subjective symptoms and diseases requiring hospital visits were examined by stress group using the Wilcoxon signed-rank test. The results showed a significant difference between groups in both subjective symptoms and diseases requiring hospital visits (*p* < 0.01).

In addition, the mean number of responses for subjective symptoms was calculated and compared by stress group. The results showed 3.61 ± 3.09 items in the low-stress group and a significantly greater number (6.21 ± 5.20 items) in the high-stress group (*p* < 0.01). Similarly, with the mean number of responses for diseases requiring hospital visits, there were 1.95 ± 1.26 items in the low-stress group and a significantly greater number (2.39 ± 1.81) in the high-stress group (*p* < 0.01).

### 3.3. Results of Contingency Table Analysis of Basic Attributes, Subjective Symptoms, and Hospital Visits by Stress Group

Table 4 shows the results of the contingency table analysis by stress group of gender, age, household composition, work hours, subjective symptoms, hospital visits, lifestyle, and QOL, for which a significant difference was found.

In the high-stress group, significantly greater proportions of subjects were women, aged 40–64 years, and in single-person households and had long work hours, subjective symptoms, a disease requiring hospital visits, insufficient sleep, smoking, and poor QOL, all of which showed significant differences (*p* < 0.01).

**Table 4:** Comparison by stress group of basic attributes, subjective symptoms/diseases, and other survey items.

Survey Item (*)	High-Stress Group	Low-Stress Group	Total	X^2^ Test	Univariate Odds Ratio
Gender					
Male (1)	2027 (7.7)	24,138 (92.3)	26,165 (100.0)	**	0.76
Female (0)	2837 (9.9)	25,864 (90.1)	28,701 (100.0)		
Age group (years)					
Old age group: 65–89 (1)	1689 (7.0)	22,286 (93.0)	23,975 (100.0)	**	0.66
Control group: 40–64 (0)	3175 (10.3)	27,716 (89.7)	30,891 (100.0)		
Household structure					
Living alone, other alone (1)	813 (10.4)	6972 (89.6)	7785 (100.0)	**	1.23
Spouse/three-generation household, parent–child household (0)	4051 (8.6)	43,030 (91.4)	47,081 (100.0)		
Work hours					
≥60 (1)	1997 (9.7)	18,587 (90.3)	20,584 (100.0)	**	1.24
<60 (0)	2239 (8.0)	25,881 (92.0)	28,120 (100.0)		
Presence of subjective symptoms					
Yes (1)	2997 (15.1)	16,827 (84.9)	19,824 (100.0)	**	3.17
No (0)	1838 (5.3)	32,811 (94.7)	34,649 (100.0)		
Presence of hospital visits (disease)					
Yes (1)	3046 (10.2)	26,731 (89.8)	29,777 (100.0)	**	1.46
No (0)	1790 (7.2)	23,046 (92.8)	24,836 (100.0)		
Sufficient sleep					
No (1)	2499 (20.8)	9490 (79.2)	11,989 (100.0)	**	4.59
Yes (0)	2281 (5.4)	39,811 (94.6)	42,092 (100.0)		
Smoking					
With smoking habit (1)	1017 (10.2)	8915 (89.2)	9932 (100.0)	**	1.22
No (0)	3779 (8.5)	40,514 (91.5)	44,293 (100.0)		
Health perception: QOL					
Poor (1)	2194 (27.5)	5787 (72.5)	7981 (100.0)	**	6.31
Good/regular (0)	2639 (5.7)	43,976 (94.3)	46,615 (100.0)		

* Set as explanatory variables for the binomial logistic regression. (N [%]; **: *p* < 0.01).

### 3.4. Results of Contingency Table Analysis of Stress Groups with Oral Symptoms and Oral Disease

Table 5 shows the results of the contingency table analysis of oral symptoms (aggregated by three symptoms) and oral disease by stress group. With dental symptoms, the contingency table analysis of the three symptoms, either separately or aggregated, showed significant differences between groups (*p* < 0.01). There was a slightly higher proportion of subjects with tooth disease in the high-stress group, but this difference was not significant.

**Table 5:** Comparison by stress group of presence of dental symptoms and tooth disease.

Survey Item	Response (*)	High-Stress Group (%)	Low-Stress Group (%)	Total (%)	X^2^ Test	Univariate Odds Ratio
Total						
Dental symptoms	Yes (1)	682 (21.3)	2517 (78.7)	3199 (100.0)	**	1.67
	No (0)	2315 (13.9)	14,310 (86.1)	16,625 (100.0)		
(Repeated)						
Toothache	Yes	245 (23.3)	806 (76.7)	1051 (100.0)	**	1.76
	No	2752 (14.7)	16,827 (85.3)	18,773 (100.0)		
Bleeding/swollen gums	Yes	295 (22.3)	1030 (77.7)	1325 (100.0)	**	1.67
	No	2702 (15.1)	15,797 (84.9)	18,499 (100.0)		
Difficulty chewing	Yes	330 (23.5)	1073 (76.5)	1403 (100.0)	**	1.81
	No	2667 (14.5)	15,754 (85.5)	18,421 (100.0)		
Disease name						
Tooth disease	Yes	417 (10.7)	3475 (89.3)	3892 (100.0)	*p* = 0.297	1.06
	No	2629 (10.2)	23,256 (89.8)	25,885 (100.0)		

* Set as explanatory variables for the binomial logistic regression. (N [%]; **: *p* < 0.01).

### 3.5. Results of Contingency Table Analysis of Stress Groups with Systemic Symptoms

Table 6 shows the results of the comparison of systemic symptoms by stress group, with symptoms that had significant differences shown in order from the highest response rate.

There was a tendency toward a higher proportion of subjects in the high-stress group with musculoskeletal symptoms, which had the highest response rates, and several other subjective symptoms. Subjective symptoms with response rates ranking in the top 10 and with proportions of >20% in the high-stress group were itchy eyes (22.3%), feeling listless (33.0%), numbness of limbs (22.0%), and difficulty seeing things (23.2%).

In addition, symptoms that ranked lower than the top 10 for response rate but had significant differences in response rate by stress group are shown in the lower part of Table 5; these include symptoms with high univariate ORs. The symptoms with an OR of >2 were irritability (6.86), sleeplessness (3.80), feeling listless (3.58), and forgetfulness (2.29).

The proportion of subjects in the high-stress group with irritability was extremely high, at 49.5%, and sleeplessness was also high, at 35.9%. These were the only symptoms with proportions of >30% in the high-stress group.

All of the subjective symptoms shown in Table 5 show a significant difference in the proportion of subjects between the high-stress and control groups (*p* < 0.01).

**Table 6:** Comparison by stress group of presence of systemic symptoms (top 10 response rates and high-odds-ratio items are shown).

Condition	Response (*)	High-Stress Group (%)	Low-Stress Group (%)	Total (%)	X^2^ Test	Univariate Odds Ratio
Lower back pain	Yes (1)	1306 (17.3)	6253 (82.7)	7559 (100.0)	**	1.31
	No (0)	1691 (13.8)	10,574 (86.2)	12,265 (100.0)		
Stiff shoulders	Yes (1)	1128 (18.4)	4999 (81.4)	6127 (100.0)	**	1.43
	No (0)	1869 (13.6)	11,828 (86.4)	13,697 (100.0)		
Joint pain in hands and feet	Yes (1)	795 (18.1)	3602 (81.9)	4397 (100.0)	**	1.32
	No (0)	2202 (14.3)	13,225 (85.7)	15,427 (100.0)		
Blurred vision	Yes (1)	711 (22.3)	2483 (77.7)	3194 (100.0)	**	1.80
	No (0)	2286 (13.7)	14,344 (86.3)	16,630 (100.0)		
Cough or phlegm	Yes (1)	539 (18.79)	2329 (81.21)	2868 (100.0)	**	1.37
	No (0)	2458 (14.50)	14,498 (85.50)	16,956 (100.0)		
Feeling listless	Yes (1)	943 (33.0)	1912 (67.0)	2855 (100.0)	**	3.58
	No (0)	2054 (12.1)	14,915 (87.9)	16,969 (100.0)		
Numbness of arms or legs	Yes (1)	603 (22.0)	2135 (78.0)	2738 (100.0)	**	1.73
	No (0)	2394 (14.0)	14,692 (86.0)	17,086 (100.0)		
Difficulty seeing things	Yes (1)	631 (23.2)	2094 (76.8)	2725 (100.0)	**	1.87
	No (0)	2366 (13.8)	14,733 (86.2)	17,099 (100.0)		
Frequent urination	Yes (1)	477 (18.6)	2086 (81.4)	2563 (100.0)	**	1.34
	No (0)	2520 (14.6)	14,741 (85.4)	17,261 (100.0)		
Blocked/runny nose	Yes (1)	477 (19.4)	1988 (80.7)	2465 (100.0)	**	1.41
	No (0)	2520 (14.9)	14,839 (85.5)	17,359 (100.0)		
Irritability	Yes (1)	693 (49.5)	707 (50.5)	1400 (100.0)	**	6.86
	No (0)	2304 (12.5)	16,120 (87.5)	18,424 (100.0)		
Sleeplessness	Yes (1)	708 (35.9)	1264 (64.1)	1972 (100.0)	**	3.80
	No (0)	2289 (12.8)	15,563 (87.2)	17,852 (100.0)		
Forgetfulness	Yes (1)	610 (26.5)	1692 (73.5)	2302 (100.0)	**	2.29
	No (0)	2387 (13.6)	15,135 (86.4)	17,522 (100.0)		
Constipation	Yes (1)	549 (24.3)	1711 (75.7)	2260 (100.0)	**	1.98
	No (0)	2448 (13.9)	15,116 (86.1)	17,564 (100.0)		
Ringing in the ears	Yes (1)	440 (19.3)	1835 (80.7)	2275 (100.0)	**	1.41
	No (0)	2557 (14.6)	14,992 (85.4)	17,549 (100.0)		
Difficulty hearing	Yes (1)	455 (19.0)	1935 (81.0)	2390 (100.0)	**	1.37
	No (0)	2542 (14.6)	14,892 (85.4)	17,434 (100.0)		

Upper section: symptoms with top 10 response rates; lower section: other symptoms with high odds ratios. * Set as explanatory variables for the binomial logistic regression. (**: *p* < 0.01).

### 3.6. Results of Contingency Table Analysis of Stress Groups with Systemic Diseases

Table 7 shows the results of contingency table analysis of systemic disease by stress group for the systemic diseases with the top-ranked response rates and largest ORs.

**Table 7:** Comparison of presence of systemic disease (top 10 response rates and high-odds-ratio items are shown).

Disease Name	Response (*)	High-Stress Group (%)	Low-Stress Group (%)	Total (%)	X^2^ Test	Univariate Odds Ratio
High blood pressure	Yes (1)	867 (8.1)	9824 (91.9)	10,691 (100.0)	**	0.68
	No (0)	2179 (11.4)	16,907 (88.6)	19,086 (100.0)		
Dyslipidemia	Yes (1)	416 (8.8)	4293 (91.2)	4709 (100.0)	**	0.82
	No (0)	2630 (10.5)	22,438 (89.5)	25,068 (100.0)		
Eye disease	Yes (1)	457 (10.6)	3870 (89.4)	4327 (100.0)	*p* = 0.451	1.04
	No (0)	2589 (10.2)	22,861 (89.8)	25,450 (100.0)		
Diabetes	Yes (1)	406 (9.4)	3912 (90.6)	4318 (100.0)	*p* = 0.559	0.90
	No (0)	2640 (10.4)	22,819 (89.6)	25,459 (100.0)		
Lumbago	Yes (1)	493 (13.2)	3264 (86.8)	3762 (100.0)	**	1.41
	No (0)	2548 (9.8)	23,467 (90.2)	26,015 (100.0)		
Stiff shoulders	Yes (1)	276 (15.3)	1528 (84.7)	1804 (100.0)	**	1.64
	No (0)	2770 (9.9)	25,203 (90.1)	27,973 (100.0)		
Joint disease	Yes (1)	227 (13.6)	1447 (86.4)	1674 (100.0)	**	1.41
	No (0)	2819 (10.0)	25,284 (90.0)	28,103 (100.0)		
Other disease	Yes (1)	244 (15.9)	1291 (84.1)	1535 (100.0)	**	1.72
	No (0)	2802 (9.9)	25,440 (90.1)	28,242 (100.0)		
Osteoporosis	Yes (1)	195 (13.2)	1278 (86.8)	1473 (100.0)	**	1.36
	No (0)	2851 (10.1)	25,453 (89.9)	28,304 (100.0)		
Angina/cardiac infarction	Yes (1)	181 (12.3)	1287 (87.7)	1468 (100.0)	**	1.24
	No (0)	2865 (10.1)	25,444 (89.9)	28,309 (100.0)		
Depression/other mental disease	Yes (1)	489 (42.7)	656 (57.3)	1145 (100.0)	**	7.60
	No (0)	2557 (8.9)	26,075 (91.1)	28,632 (100.0)		
Allergic rhinitis	Yes (1)	202 (14.3)	1211 (85.7)	1413 (100.0)	**	1.50
	No (0)	2844 (10.0)	25,520 (90.0)	28,364 (100.0)		
Other skin disease	Yes (1)	165 (13.0)	1102 (87.0)	1267 (100.0)	**	1.33
	No (0)	2881 (10.1)	25,629 (89.9)	28,510 (100.0)		
Other cardiovascular disease	Yes (1)	167 (12.4)	1176 (87.6)	1343 (100.0)	**	1.26
	No (0)	2879 (10.1)	25,555 (89.9)	28,434 (100.0)		
Gastric/intestinal disease	Yes (1)	145 (12.2)	1041 (87.8)	1186 (100.0)	*	1.23
	No (0)	2910 (10.1)	25,690 (89.9)	28,591 (100.0)		

Upper section: diseases with top 10 response rates; lower section: other diseases with high odds ratios (**: *p* < 0.01; *: *p* < 0.05). * Set as explanatory variables for the binomial logistic regression.

The diseases with the top-ranked response rates included diseases that are common among older adults, such as high blood pressure. Additionally, there were also diseases such as lumbago and shoulder stiffness, which corresponded to top-ranked subjective symptoms.

The upper part of Table 6 shows the diseases with the top 10 ranked response rates. For each of the seven diseases other than high blood pressure, dyslipidemia, and diabetes, the high-stress group showed a higher proportion of subjects with the disease than without. For all diseases other than eye disease and diabetes, there was a significant difference in the proportion in the high-stress group (*p* < 0.01).

The lower part of Table 6 shows the diseases with high ORs. A high proportion of subjects with depression/other mental disease (42.7%) were in the high-stress group. With all other diseases, the proportion in the high-stress group showed a significant difference (stomach/duodenal disease: *p* < 0.05; all others: *p* < 0.01). The univariate OR was highest for depression/other mental disease, at 7.60, and the OR was <2 for all other diseases. High blood pressure, hyperlipidemia, and diabetes showed univariate ORs of <1.

### 3.7. Results of Binomial Logistic Regression with Stress Group and QOL as Dependent Variables

The symptoms and diseases that demonstrated an association with the stress group in the contingency table analysis were analyzed using binomial logistic regression, with the stress group as the dependent variable (Model 1) and QOL as the dependent variable (Model 2). The adjusted OR for each explanatory variable was obtained, and a comparison was performed. The number of valid cases (subjects with no missing responses for all analysis items) for the binomial logistic regression was over 10,000 for the analysis in each model.

Table 8 shows the analysis results for Models 1 and 2. In Model 1, the variables with large adjusted ORs were irritability (3.34), depression/other mental disease (3.31), and QOL (3.17), all of which had values > 3. The 12 variables with significant adjusted ORs included subjective symptoms, such as feeling listless, sleeplessness, dental symptoms, constipation, and itchy eyes. The health-related behavior of undergoing health checks also had a significant OR (1.34).

In Model 2, the stress group showed the largest adjusted OR (3.14). Sixteen explanatory variables had significant adjusted ORs, more than those in Model 1. Compared with Model 1, Model 2 showed a trend toward a greater number of fatigue-related symptoms (feeling listless) and diseases (e.g., lumbago). Other than the stress group, the variables with an adjusted OR ≥ 2 were feeling listless (2.44), sleep sufficiency (2.22), and numbness of limbs (2.00).

The explanatory variables with significant adjusted ORs in both models included work hours, constipation, and eye symptoms.

Regarding the accuracy of the analysis, the coefficient of determination (Cox–Snell) was small for both models, but this was probably due to the use of a census approach for the purpose of descriptive epidemiological analysis. Because explanatory variables with high adjusted ORs were found, no variables were found to be linearly combined, the significance of the regression formula (*p* < 0.01) was confirmed, and the models were confirmed as valid for analysis.

## 4. Discussion

This study’s results indicate the existence of subjective symptoms and diseases that appear to be associated with stress in old age. Significantly, this finding was obtained from anonymized Japanese national survey statistics.

Tsuchiya et al. [24] estimated the 12-month prevalence of common mental disorders in Japan, which include anxiety and mood disorders, to be 9.1%. This figure is close to the proportion of subjects in the high-stress group in the present study (8.9%). Kessler et al. [25] used a score of 13 as the screening standard for the K6 target population, which corresponds to severe depression or a mental disorder. However, they stated that the screening level should be considered depending on the subject’s characteristics, such as gender. Moreover, in Japan, a score of 10 to 12 is used as the standard for suspected depression or anxiety disorders in official surveys. In previous studies, we divided patients into groups based on a 10-point scale, which was set with the assumption of preventive early intervention. This was set with reference to the score distribution in the target population, but we would like to conduct a further examination of the accuracy of detecting signs of a worsening trend.

The approach of viewing stress as a cause or risk factor of disease is widely accepted in Japan and worldwide, and this is reflected in the DSM-5 (Text Revision) of the American Psychiatric Association [26]. The International Classification of Diseases, 11th Revision (ICD-11), which was published in 2022, sets out “disorders specifically associated with stress” as a classification of diseases with stress as a major factor [27]. The effects of stress have also been highlighted in dental care, and the importance of stress as a risk factor has been indicated in treatment guidelines for periodontal disease [28].

Selye [29] examined the effects of stress on the body, showing that there are nonspecific stress reactions that occur systemically, regardless of the type of stress stimulus, and that physiologically, these are centered around the adrenocorticotropic hormone–adrenal cortex hormone system [30]. The present results confirm that a wide range of subjective symptoms and diseases, from local to systemic, require a hospital visit and are associated with the stress group, and that the mean numbers of symptoms and diseases were significantly greater in the high-stress group. These results may reflect the concept of allostasis [31] and can be interpreted as an adaptive phenomenon, whereby multiple brain–body regulatory systems mutually interact to cope with physical and mental disorders in response to the environment.

In addition, the present multivariate analysis results confirmed the strength of the association between QOL and stress, suggesting that stress measures should be prioritized in future interventions aimed at improving QOL. The WHO defines QOL as “an individual’s perception of their position in life in the context of the culture and value systems in which they live and in relation to their goals, expectations, standards and concerns” [32]. This study targeted age groups for analysis, and the results suggest the importance of enhancing the functions of comprehensive community care systems to extend healthy longevity and improve QOL in later life. Such systems would need to strengthen measures to improve independence among older adults, such as maintenance of physical function, economic stability, and provision of a suitable living environment, while also maintaining comfort and convenience and focusing on mental purpose in life, sense of fulfillment, and social connections [33]. Furthermore, the size of the influence of fatigue-related symptoms and long working hours points to the importance of measures for stress as part of occupational health [34], while the risk of isolation points to the need for a community-related approach.

Old age has always been seen as a period when individuals are plagued by chronic pain, such as lower back pain and limb pain, as a result of organic and functional disorders of the joints, nerves, muscles, blood vessels, and other tissue types, which can often lead to stress [35]. The results of the analysis at each stage also support the importance view. Chronic pain can lead to motor and functional decline, and persistent pain can cause anxiety about frailty [36], forcing the individual to become dependent on nursing care. Besides pain, a wide variety of other symptoms carry the risk of visits to multiple types of medical institutions and the excessive use of medication, which can intensify stress. Therefore, it is important to clarify the causal structure.

Some researchers started using the word “multimorbidity”, which is defined as “the co-existence of two or more long-term conditions in an individual”. Multimorbidity has become one of the most important topics in recent primary care because of its clinical significance [37,38]. Lin et al. [39] reported that individuals with multimorbidity face higher risks of depression, anxiety disorders, and stress states based on epidemiological survey results in China, stating that improving multiple chronic conditions is linked to improvements in mental disorders. The results of this study also show that the average number of subjective symptoms and illnesses in the high-stress group was significantly higher than in the low-stress group, indicating a relationship between stress and multimorbidity.

The coexistence of multimorbidity has become a significant challenge in the fields of health and mental healthcare worldwide, but elucidating its underlying causes is complex and difficult. Enoki et al. [40] used the same Comprehensive Survey of Living Conditions as the present study to analyze the causal relationship of stressors in middle age and showed the complexity of this relationship. Further analysis of stress factors is, therefore, important. The present results also demonstrated that fatigue-related symptoms, such as feeling listless and lower back pain, as well as high blood pressure, which is influenced by nervous tension, were more common in the high-stress group. This indicates the existence of specific symptoms and diseases that are susceptible to stress. Therefore, a detailed analysis of the process by which stress affects these conditions is needed.

A focus on dental-related health problems is also needed from the perspective of reduced nutrition in older adults [41]. The results of the contingency table analysis in this study confirmed an association between dental symptoms and stress group, but no association with tooth disease was found, and the results of the binomial logistic regression showed different trends for subjective symptoms and diseases requiring hospital visits. There are probably different factors behind this difference in trends, but a detailed investigation of the reasons for seeking dental treatment is still needed.

Furthermore, the binomial logistic regression confirmed the effects of work hours and household structure. In 1998, the WHO proposed the concept of Social Determinants of Health [42], which is the idea that health problems and diseases are the result of biological factors as well as social, environmental, and geographical factors, including the employment and household environment variables that were used in the present analysis. The Social Determinants of Health include items that cannot readily be changed, but in Japan, the importance of measures that focus on social capital has been identified [43].

The limitations of this study can be broadly divided into those related to the data and research design and those related to the influence of methodological and analytical factors. Limitations related to the data and study design include the use of data from the Comprehensive Survey of Living Conditions, which only provides a cross-sectional view of short-term subjective symptoms reported from the “past few days.” This makes it difficult to gain a picture of acute symptoms that tend to disappear within a short time. The use of the K6 to evaluate stress has also been criticized [44], as this scale may not be sufficient to investigate symptoms and diseases that require an emphasis on gender or age.

Furthermore, the effects of stress as a factor causing disease can only be grasped in a cross-sectional and self-report bias because methodological analysis was performed by comparing ORs calculated from the results of a cross-sectional questionnaire survey with numerous items. Thus, further research is needed to clarify the causal relationships in detail.

However, a major strength of this study was that it was possible to secure far more subjects than similar studies conducted in the past by using the massive dataset of the Japanese Comprehensive Survey of Living Conditions for analysis [45]. This study was able to make use of societal variables from the household questionnaire, and the survey items included questions relating to specialized subjects, such as dental care, as well as the details of health checkups. This enabled the construction of diverse analysis models, which will allow analyses to be conducted from different perspectives in the future.

The authors intend to investigate the process by which stress affects the mind and body further by narrowing down the analysis items on the basis of the present results and conducting analyses using models with high degrees of conformity.

## 5. Conclusions

In this study, anonymized data of middle-aged and older respondents from the 2019 Comprehensive Survey of Living Conditions were used, and subjects were classified into high- and low-stress groups based on the K6. A contingency table analysis of these groups with subjective symptoms, diseases requiring hospital visits, and other survey items was then performed. Additionally, adjusted odds ratios were compared using binary logistic regression analysis.

The results showed that the stress group was associated with symptoms such as irritability and dental symptoms and with diseases such as depression/other mental diseases. The taxa of stress groups were also shown to be associated with household structure, work hours, and health checkups. These results indicate the existence of specific symptoms, diseases, and environmental factors associated with high stress. These findings will be useful for future interventions to prevent stress in old age, as well as healthcare and medical measures, which would also include dental care.

Variables with large adjusted odds ratios obtained using QOL as the dependent variable included the stress group (3.14), feeling listless (2.44), sleep rest adequacy (2.22), numbness of arms or legs (2.00), and depression/other mental disease (1.85). A strong association was also found between the stress group and QOL, suggesting that measures to improve QOL in later life should focus on clarifying the effects of stress and establishing measures to mitigate its impact.

## Figures and Tables

**Table 1:** Designation of comparison groups based on responses to the 6-item version of the Kessler Psychological Distress Scale.

Kessler Psychological Distress Scale (K6) Total Points	Frequency (N)	%		Stress Group
0	21,692	39.5	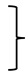	
1–3	15,119	27.6		Low-stress group
4–6	8700	15.9		*n* = 50,002
7–9	4491	8.2		
10–12	2981	5.4	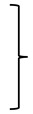	
13–15	979	1.8		
16–18	493	0.9		High-stress group
19–21	229	0.4		*n* = 4864
22–24	182	0.3		
Total	54,866	100.0		

**Table 2:** Comparison of response rates for symptoms by K6 group classification (up to 10th place shown).

Ranking of Symptoms	1st	2nd	3rd	4th	5th	6th	7th	8th	9th	10th
High-stress group	Lower back pain	Stiff shoulders	Feeling listless	Joint pain in hands and feet	Blurred vision	Sleeplessness	Irritability	Dental conditions	Difficulty seeing things	Forgetfulness
N (%)	1306 (43.6)	1128 (37.6)	943 (31.5)	795 (26.5)	711 (23.7)	708 (23.6)	693 (23.1)	682 (22.8)	631 (21.1)	610 (20.4)
Low-stress group	Lower back pain	Stiff shoulders	Joint pain in hands and feet	Dental conditions	Blurred vision	Cough or phlegm	Numbness of arms or legs	Difficulty seeing things	Frequent urination	Blocked nose
N (%)	6253 (37.2)	4999 (29.7)	3602 (21.4)	2517 (15.0)	2483 (14.8)	2329 (13.8)	2135 (12.7)	2094 (12.4)	2086 (12.4)	1988 (11.8)
Total	Lower back pain	Stiff shoulders	Joint pain in hands and feet	Blurred vision	Dental conditions	Cough or phlegm	Feeling listless	Numbness of arms or legs	Difficulty seeing things	Frequent urination
N (%)	8159 (41.2)	6516 (32.9)	4732 (23.9)	3491 (17.6)	3475 (17.5)	3102 (15.6)	3053 (15.4)	2971 (15.0)	2963 (14.9)	2818 (14.2)

**Table 3:** Comparison of response rates for diseases by K6 group classification (up to 10th place shown).

Ranking of Diseases	1st	2nd	3rd	4th	5th	6th	7th	8th	9th	10th
High-stress group	High blood pressure	Lumbago	Depression/other mental disease	Eye disease	Tooth disease	Dyslipidemia	Diabetes	Stiff shoulders	Other	Joint disease
N (%)	867 (28.5)	498 (16.3)	489 (16.1)	457 (15.0)	417 (13.7)	416 (13.7)	406 (13.3)	276 (9.1)	244 (8.0)	227 (7.5)
Low-stress group	High blood pressure	Dyslipidemia	Diabetes	Eye disease	Tooth disease	Lumbago	Stiff shoulders	Joint disease	Other	Angina/cardiac infarction
N (%)	9824 (36.8)	4293 (16.1)	3912 (14.6)	3870 (14.5)	3475 (13.0)	3264 (12.2)	1528 (5.7)	1447 (5.4)	1291 (4.8)	1287 (4.8)
Total	High blood pressure	Dyslipidemia	Eye disease	Diabetes	Tooth disease	Lumbago	Stiff shoulders	Joint disease	Osteoporosis	Other
N (%)	11,448 (38.4)	4962 (16.7)	4680 (15.7)	4675 (15.7)	4111 (13.8)	4106 (13.8)	1970 (6.6)	1802 (6.1)	1631 (5.5)	1605 (5.4)

**Table 8:** Binomial logistic regression with stress group and QOL as dependent variables.

Analysis Model	Model 1			Model 2		
No. of Valid Cases	12,418			12,418		
Dependent Variable (Evaluation)	Stress Group (K6 Total Score ≥ 10: 1; <10: 0)			QOL (Poor and Fairly Poor: 1; Regular, Fairly Good, and Good: 0)		
Explanatory Variables	Variable	Adjusted Odds Ratio	95% CI	Variable	Adjusted Odds Ratio	95% CI
	Irritability	3.341 **	2.80–3.99	Stress group	3.148 **	2.79–3.55
	Depression/other mental disease	3.319 **	2.74–4.02	Feeling listless	2.443 **	2.16–2.76
	QOL	3.173 **	2.82–3.57	Sleep rest adequacy	2.223 **	2.03–2.44
	Sleep rest adequacy	2.264 **	2.01–2.55	Numbness of arms or legs	2.004 **	1.79–2.25
	Feeling listless	1.482 **	1.29–1.71	Depression/other mental disease	1.850 **	1.53–2.24
	Sleeplessness	1.353 **	1.15–1.59	Lumbago	1.475 **	1.30–1.67
	Health checkups	1.341 **	1.19–1.52	Work hours	1.415 **	1.29–1.55
	Dental conditions	1.243 **	1.06–1.46	Constipation	1.361 **	1.19–1.55
	Constipation	1.226 *	1.04–1.44	Difficulty hearing	1.343 **	1.17–1.54
	Household structure	1.191 *	1.02–1.38	Hand and foot joint pain	1.298 **	1.17–1.44
	Blurred vision	1.187 *	1.02–1.38	Joint disease	1.286 **	1.11–1.52
	Work hours	1.180 **	1.04–1.34	Health checkups	1.283 **	1.17–1.41
				Gender ^¶^	1.243 **	1.14–1.36
				Sleeplessness	1.232 **	1.07–1.42
				Cough or phlegm	1.197 **	1.06–1.35
				Difficulty seeing things	1.162 *	1.03–1.31
Coefficient of determination	R^2^ (Cox–Snell)	0.16		R^2^ (Cox–Snell)	0.17	

Gender and other variables from Table 4 are included as moderator variables. ^¶^ for calculation of adjusted odds ratios. (**: *p* < 0.01; *: *p* < 0.05). Only items with an adjusted odds ratio >1 that were significant are displayed.

## Data Availability

The original contributions presented in this study are included in the article. Further inquiries can be directed to the corresponding author.

## References

[1] Karam G., Itani L., Fayyad J., Karam A., Mneimneh Z., Karam E. (2016). Prevalence, Correlates, and Treatment of Mental Disorders among Lebanese Older Adults: A National Study. Am. J. Geriatr. Psychiatry.

[2] Ministry of Health, Labour and Welfare Annual Health, Labour and Welfare Report 2024. https://www.mhlw.go.jp/english/wp/index.html.

[3] Kawakami N., Haratani T. (2019). Epidemiology of job stress and health in Japan: Review of current evidence and future direction. Ind. Health.

[4] Väranik P. (2012). Suicide in the World. Int. J. Environ. Res. Public Health.

[5] World Health Organization Preventing Suicide: A Global Imperative. https://www.who.int/publications/i/item/9789241564779.

[6] Hammen C. (2005). Stress and depression. Annu. Rev. Clin. Psychol..

[7] Salleh M.R. (2008). Life event, stress and illness. Malays. J. Med. Sci..

[8] Coelho J.M.F., Miranda S.S., Cruz S.S.D., Trindade S.C., Passos-Soares J.D.S., Cerqueira E.M.M., Costa M.C.N., Figueiredo A.M.G., Hintz A.M., Barreto M.L. (2020). Is there association between stress and periodontitis?. Clin. Oral. Investig..

[9] Hara K., Nagata T., Matoba M., Miyazaki T. (2025). The Impact of Productivity Loss from Presenteeism and Absenteeism on Mental Health in Japan. J. Occup. Environ. Med..

[10] World Health Organization Constitution: Who Remains Firmly Committed to the Principles Set Out in the Preamble to the Constitution. https://www.who.int/about/governance/constitution.

[11] Albertoni C.C., Deny O., Benard V.P., Guissard C., Paupert J., Vaysse F., Marty M., Casteilla L., Monsarrat P., Kemoun P. (2024). The oral organ: A new vision of the mouth as a whole for a gerophysiological approach to healthy aging. Ageing Res. Rev..

[12] Sato A., Eguchi E., Hayashi F., Funakubo N., Okada T., Kiyama M., Imano H., Ohira T. (2025). A prospective study of the association between lifestyle and the risk of depressive symptoms. J. Affect. Disord..

[13] Niizato K.A. (2020). consideration of the effects of aging on psychosomatic symptoms in the elderly. Rinsho Shinkeigaku.

[14] Chauhan A., Jain C.K. (2024). Psychosomatic Disorder: The Current Implications and Challenges. Cardiovasc. Hematol. Agents Med. Chem..

[15] Glaser R., Kiecolt-Glaser J.K. (2005). Stress-induced immune dysfunction: Implications for health. Nat. Rev. Immunol..

[16] Versteeg H., Spek V., Pedersen S.S., Denollet J. (2012). Type D personality and health status in cardiovascular disease populations: A meta-analysis of prospective studies. Eur. J. Prev. Cardiol..

[17] Yaribeygi H., Panahi Y., Sahraei H., Johnston T.P., Sahebkar A. (2017). The impact of stress on body function: A review. EXCLI J..

[18] Komatsuzaki A., Ono S. (2020). Study of the Effects of Recognition of Stress on Symptoms and Regular Hospital Visits: An Analysis from Japanese National Statistics. Healthcare.

[19] Johnson J., Ellis R.S., Janes G., Mills T., Budworth L., Atkinson L., Harrison R. (2020). Can we prepare healthcare professionals and students for involvement in stressful healthcare events? A mixed-methods evaluation of a resilience training intervention. BMC Health Serv. Res..

[20] Helmuth B., Broitman B.R., Yamane L., Gilman S.E., Mach K., Mislan K.A.S., Denny M.W. (2010). Organismal climatology: Analyzing environmental variability at scales relevant to physiological stress. J. Exp. Biol..

[21] Fu Q., Li L., Li Q., Wang J. (2025). The effects of physical activity on the mental health of typically developing children and adolescents: A systematic review and meta-analysis. BMC Public Health.

[22] Komatsuzaki A., Ono S., Mitomi K., Arashi K., Enoki Y., Seino K., Komatsuzaki N., Ikeda Y. (2024). A Study of the Factors Impeding Proper Dietary Habits: An Investigation Using the Japanese Comprehensive Survey of Living Conditions. Clin. Pract..

[23] Ministry of Health, Labour and Welfare Ethical Guidelines for Medical and Health Research Involving Human Subjects. https://www.mhlw.go.jp/file/06-Seisakujouhou-10600000-Daijinkanboukouseikagakuka/0000080278.pdf.

[24] Tsuchiya M., Kawakami N., Ono Y., Nakane Y., Nakamura Y., Fukao A., Tachimori H., Iwata N., Uda H., Nakane H. (2012). Impact of mental disorders on work performance in a community sample of workers in Japan: The World Mental Health Japan Survey 2002–2005. Psychiatry Res..

[25] Kessler R.C., Barker P.R., Colpe L.J., Epstein J.F., Gfroerer J.C., Hiripi E., Howes M.J., Normand S.L.T., Manderscheid R.W., Walers E.E. (2003). Screening for serious mental illness in the general population. Arch. Gen. Psychiatry..

[26] American Psychiatric Association (2022). Diagnostic and Statistical Manual of Mental Disorders.

[27] World Health Organization Who Releases 2025 Update to the International Classification of Diseases (ICD-11). https://www.who.int/news/item/14-02-2025-who-releases-2025-update-to-the-international-classification-of-diseases-(icd-11).

[28] The Japanese Society of Periodontology JSP Clinical Practice Guidelines for the Periodontal Treatment 2022. https://www.perio.jp/publication/upload.file/guideline_perio_2022.pdf.

[29] Selye H. (1956). The Stress of Life.

[30] Kawata M. (1995). Roles of steroid hormones and their receptors in structural organization in the nervous system. Neurosci. Res..

[31] McEwen B.S., Stellar E. (1993). Stress and the individual. Mechanisms leading to disease. Arch. Intern. Med..

[32] The WHO QOL Group (1995). The Health Organization World Quality of Life assessment (WHOQOL): Position paper from the World Health Organization. Soc. Sci. Med..

[33] Song P., Tang W. (2019). The community-based integrated care system in Japan: Health care and nursing care challenges posed by super-aged society. Biosci. Trends.

[34] Dollard M., Loh M.Y. (2023). Psychosocial safety climate in Japanese workplaces. J. Occup. Health.

[35] Takegami N., Akeda K., Yamada J., Nishimura A., Suda A. (2024). Association between low back pain and psychological stress response in a Japanese population-based study. J. Orthop. Sci..

[36] Tanimoto Y., Watanabe M., Sun W., Sugiura Y., Tsuda Y., Kimura M., Hayashida I., Kusabiraki T., Kono K. (2012). Association between sarcopenia and higher-level functional capacity in daily living in community-dwelling elderly subjects in Japan. Arch. Gerontol Geriatr..

[37] Zhou Y., Kivimaki M., Lunstad J.H., Yan L., Zhang Y., Wang H., Wang S., Xu X. (2025). Stressful life events in childhood and adulthood and risk of physical, psychological and cognitive multimorbidities: A multicohort study. eClinicalMedicine.

[38] Klopack E.T. (2023). Chronic Stress and Latent Virus Reactivation: Effects on Immune Aging, Chronic Disease Morbidity, and Mortality. J. Gerontol. B. Psychol. Sci. Soc. Sci..

[39] Lin H., Xiao S., Shi L., Zheng X., Xue Y., Yun Q., Ouyang P., Wang D., Zhu H., Zhang C. (2021). Impact of Multimorbidity on Symptoms of Depression, Anxiety, and Stress in Older Adults: Is There a Sex Difference?. Front. Psychol..

[40] Enoki Y., Komatsuzaki N., Mitomi K., Seino K., Sekiguchi H., Komatsuzaki A., Ono S. (2024). A casual structural model for the effects of stress on physical and mental health: A discussion based on anonymized date from the Comprehensive Survery of Living Conditions. J. Nippon. Health Sci..

[41] Kikuchi H., Komatsuzaki A., Ono S., Sirono M., Motoi S., Iguchi A., Susuga M. (2021). Factors Affecting Dietary Improvements in Elderly Residents of Long-Term Care Institutions Receiving Domiciliary Dental Care. Medicines.

[42] World Health Organization Social Determinants of Health. https://www.who.int/health-topics/social-determinants-of-health#tab=tab_1.

[43] Tsubokawa T., Shobugawa Y., Iguchi S., Suzuki T., Watanabe M., Saito R., Kondo K. (2022). Do Community Social Capital and Built Environment Associate with Homebound in Older Adults? The JAGES Niigata Study. J. Epidemiol..

[44] Mewton L., Kessler R.C., Slade T., Hobbs M., Brownhill L., Birrell L., Tonks Z., Teesson M., Newton N., Chapaman C. (2016). The psychometric properties of the Kessler Psychological Distress Scale (K6) in a general population sample of adolescents. Psychol. Assess..

[45] Kurimura S., Nakaya N., Ohmori-Matsuda K., Shimazu T., Kikuchi N., Kakizaki M., Sone T., Sato F., Nagai M., Sugawara Y. (2009). Factors associated with psychological distress in a community-dwelling Japanese population: The Ohsaki Cohort 2006 Study. J. Epidemiol..

